# Scaling-up Engineering Biology for Enhanced Environmental Solutions

**DOI:** 10.1021/acssynbio.4c00292

**Published:** 2024-06-21

**Authors:** Francis Hassard, Thomas P. Curtis, Gabriela C. Dotro, Peter Golyshin, Tony Gutierrez, Sonia Heaven, Louise Horsfall, Bruce Jefferson, Davey L. Jones, Natalio Krasnogor, Vinod Kumar, David J. Lea-Smith, Kristell Le Corre Pidou, Yongqiang Liu, Tao Lyu, Ronan R. McCarthy, Boyd McKew, Cindy Smith, Alexander Yakunin, Zhugen Yang, Yue Zhang, Frederic Coulon

**Affiliations:** 1 Cranfield University, Bedford MK43 0AL, U.K.; 2 Newcastle University, Newcastle upon Tyne NE4 5TG, U.K.; 3 Bangor University, Gwynedd LL57 2UW, U.K.; 4 Heriot-Watt University, Edinburgh, EH14 4AS, U.K.; 5 University of Southampton, Southampton SO16 7QF, U.K.; 6 University of Edinburgh, Edinburgh EH9 3FF, U.K.; 7 University of East Anglia, Norwich NR4 7TJ, U.K.; 8 Brunel University London, Uxbridge UB8 3PH, U.K.; 9 University of Essex, Colchester, Essex CO4 3SQ, U.K.; 10 University of Glasgow, Glasgow G12 8LT, U.K.

**Figure 1:**
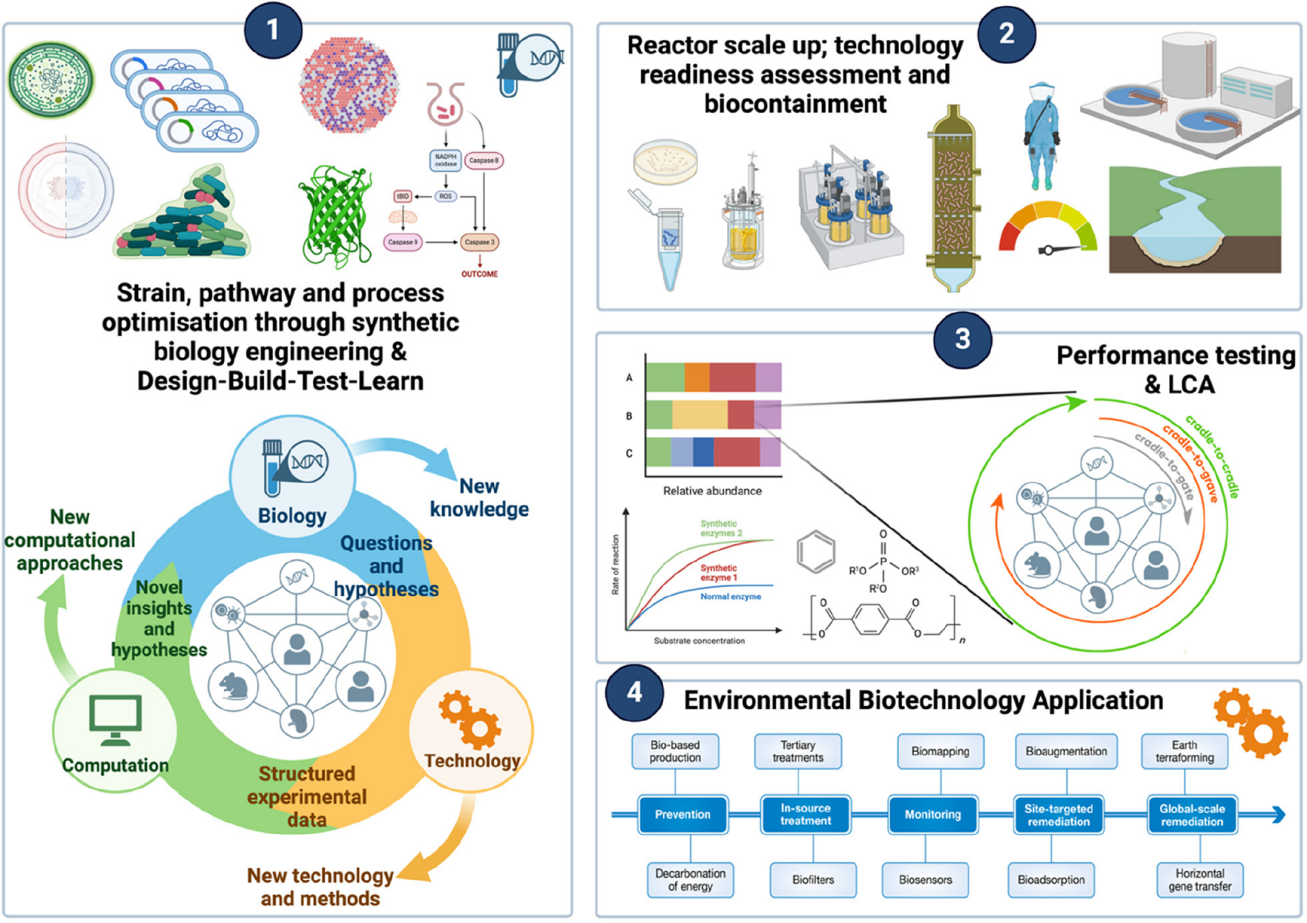
Research priorities for Synthetic Biology for Environmental Biotechnology Solutions. LCA = Life Cycle Assessment. Panel 4 is adapted from ref (1). Copyright 2018, EMBO Press.

Synthetic biology (SynBio) offers transformative solutions for addressing environmental challenges by engineering organisms capable of degrading pollutants, enhancing carbon sequestration, and valorizing waste (Figure 1). These innovations hold the potential to revolutionize bioremediation strategies, ecosystem restoration, and sustainable environmental management.^1^ Advances in SynBio, including automation, precise manipulation of genetic material,^2^ and design of semisynthetic organisms with enhanced capabilities, can improve the efficiency of microbes for eliminating pollutants such as hydrocarbons and plastics or extracting valuable resources from the environment.^3^ Genome editing technologies, such as CRISPR-Cas9, allows the editing of genomes with unprecedented accuracy, facilitating the development of organisms with desired traits or functions.^4^ Furthermore, SynBio encompasses the engineering of metabolic enzymes within organisms, leading to the design of microbial factories capable of degrading complex and persistent chemicals, and converting waste to valuable resources.^5^ These advancements also facilitate the manipulation of bacterial social behaviors, offering the capacity for tunable control at the multicellular level and engineered biofilms.^5^

Biofoundries are taking advantages of automated and high-throughput technologies to engineer biological systems, facilitating the design, construction, and testing of genetic constructs, such as synthetic genomes and minimal cells, with specific functionalities.^6^ By providing the essential infrastructure and expertise, they play a pivotal role in advancing synthetic biology and biotechnology and therefore accelerating the development of innovative environmental biotechnologies for various applications, including environmental remediation, healthcare, and industrial processes.^6^

Despite the immense promise of SynBio for environmental applications, several challenges remain, including scaling up laboratory-based experiments to real-world applications, addressing the ethical, regulatory, and public acceptance transparently, and understanding microbial interactions within engineered microbial communities, as introducing synthetic organisms or modifying existing ones could disrupt natural community dynamics and lead to unintended consequences.^7^ Thus, balancing the desired engineering goals with maintaining ecosystem stability and resilience is crucial to ensure the long-term success and sustainability of engineered microbial communities. Emerging mathematical models for metabolic transitions and interactions are invaluable tools, enhancing our understanding of both synthetic and natural consortia. These models also play a key role in strengthening iterative Design-Build-Test-Learn (DBTL) cycles and conventional bioreactor engineering approaches^8^ (Figure 1). As the scale and ambition of SynBio solutions for the environment grow, and concomitant with the increased sophistication in the DBTL cycle, there is a need to standardize chassis specification,^9^ more robustly barcode engineered strains, and more transparently track the process of cellular engineering using specialized version control systems.^10^

The UK-based Environmental Biotechnology Innovation Centre (EBIC) funded by the Biotechnology and Biological Sciences Research Council (BB/Y008332/1) serves as an Engineering Biology Hub for Environmental Solutions. EBIC is committed to enabling the responsible and safe scaling up of SynBio solutions for environmental remediation solutions with a focus on collaborative efforts and innovative approaches to foster sustainable solutions for the benefit of the society. The hub also allocated a flexible fund of nearly £2 million for open competition, specifically targeting early career researchers, aiming to advance SynBio research and cultivate the next generation of scientists.
